# ND3, ND1 and 39 kDa subunits are more exposed in the de-active form of bovine mitochondrial complex I

**DOI:** 10.1016/j.bbabio.2014.02.013

**Published:** 2014-06

**Authors:** Marion Babot, Paola Labarbuta, Amanda Birch, Sara Kee, Matthew Fuszard, Catherine H. Botting, Ilka Wittig, Heinrich Heide, Alexander Galkin

**Affiliations:** a Queen's University Belfast, School of Biological Sciences, Medical Biology Centre, 97 Lisburn Road, Belfast BT9 7BL, UK; b School of Chemistry, Biomedical Sciences Research Complex, BMS Annexe, University of St. Andrews, KY16 9ST, UK; c Functional Proteomics, SFB Core Unit, Faculty of Medicine, Goethe University Frankfurt, Theodor-Stern-Kai 7, 60590 Frankfurt am Main, Germany

**Keywords:** A/D, active/de-active transition, AH, amphipathic helix, BN-PAGE, blue native polyacrylamide gel electrophoresis, DIGE, difference gel electrophoresis, dSDS-PAGE, double SDS-PAGE, DTT, dithiothreitol, F-NHS, fluorescein-*N*-hydroxysulfosuccinimide ester, hrCN-PAGE, high resolution clear native polyacrylamide gel electrophoresis, LHON, Leber's hereditary optic neuropathy, MELAS, mitochondrial encephalomyopathy, lactic acidosis and stroke-like episodes, NADH, dihydronicotinamide adenine dinucleotide, NAI, *N*-acetylimidazole, NEM, *N*-ethylmaleimide, NHS, *N*-hydroxysuccinimide, nLC-ESI-MSMS, nano-HPLC electrospray ionisation tandem mass spectrometry, Q_1_, ubiquinone-1,2,3-dimethoxy-5-methyl-6-(3-methyl-2-butenyl)-1,4-benzoquinone, ROS, reactive oxygen species, SMP, submitochondrial particles, TMS, transmembrane segment, TNM, tetranitromethane, Complex I, NADH:ubiquinone oxidoreductase, A/D transition, Conformational change, Protein tyrosine modification, *N*-hydroxysuccinimide

## Abstract

An intriguing feature of mitochondrial complex I from several species is the so-called A/D transition, whereby the idle enzyme spontaneously converts from the active (A) form to the de-active (D) form. The A/D transition plays an important role in tissue response to the lack of oxygen and hypoxic deactivation of the enzyme is one of the key regulatory events that occur in mitochondria during ischaemia. We demonstrate for the first time that the A/D conformational change of complex I does not affect the macromolecular organisation of supercomplexes *in vitro* as revealed by two types of native electrophoresis. Cysteine 39 of the mitochondrially-encoded ND3 subunit is known to become exposed upon de-activation. Here we show that even if complex I is a constituent of the I + III_2_ + IV (S_1_) supercomplex, cysteine 39 is accessible for chemical modification in only the D-form. Using lysine-specific fluorescent labelling and a DIGE-like approach we further identified two new subunits involved in structural rearrangements during the A/D transition: ND1 (MT-ND1) and 39 kDa (NDUFA9). These results clearly show that structural rearrangements during de-activation of complex I include several subunits located at the junction between hydrophilic and hydrophobic domains, in the region of the quinone binding site. De-activation of mitochondrial complex I results in concerted structural rearrangement of membrane subunits which leads to the disruption of the sealed quinone chamber required for catalytic turnover.

## Highlights

• Supercomplex composition is not affected by mitochondrial complex I conformation.
• The D-form of complex I is selectively inhibited by tyrosine-reactive reagents.
• ND3, ND1 & 39 kDa subunits become exposed upon deactivation of complex I.

## 1 Introduction

Production of energy in most aerobic cells is provided by the joint activity of the mitochondrial respiratory chain and ATP-synthase. Most of the NADH produced in the mammalian cell, through aerobic catabolism, is oxidised by complex I or NADH:ubiquinone oxidoreductase [1], the first energy converting enzyme in mitochondria. Complex I catalyses the oxidation of matrix nucleotides by membrane ubiquinone and represents the major entry point for reducing equivalents into the respiratory chain. In contrast to the 14 subunit bacterial enzyme, mitochondrial complex I contains 30 additional ‘accessory’ subunits of which most have as yet unassigned functions [2]. Complex I redox centres (FMN and FeS clusters) are located within the hydrophilic domain of the L-shaped enzyme molecule, whilst proton translocation could be carried out by several Na^+^/H^+^ antiporter-like subunits in the membrane arm. The exact mechanisms of energy transduction between the redox reaction at the terminal cluster N2 and proton translocation in the membrane part are still not known. Recent studies [3,4] have significantly improved our understanding of the structure, the proton translocation machinery [4,5] and assembly of complex I [6] but many aspects of the enzyme's regulation require elucidation.

An intriguing feature of mitochondrial complex I from several vertebrate species and yeast is the existence of two functionally and structurally different states of the enzyme: active, A-form and de-active or dormant, D-form [7]. The enzyme in submitochondrial particles (SMP) or purified complex I as prepared is composed of a mixture of the A- and the D-forms and requires activation before kinetic measurements. In the presence of NADH and ubiquinone the D-form of complex I can be converted back to the A-form as a result of slow (4 min^− 1^ at 25 °C) catalytic turnover(s) [8]. Non-activated, heterogeneous preparations of mitochondrial membranes show a lag phase during continuous assay of NADH oxidation. After complete activation, complex I catalyses rotenone-sensitive oxidation of NADH with a fast linear rate of around 10^4^ min^− 1^ [7]. These catalytically and structurally distinct forms of the enzyme have been identified in purified preparations *in vitro*, in cells, as well as in heart *ex vivo* in ischaemia [9–13].

Until now, the only known conformational difference between the A- and D-forms of mitochondrial complex I was the exposure of cysteine 39 (Cys-39) of the mitochondrially encoded ND3 subunit in the D-form [14]. Covalent modification of this critical thiol renders the D-form sensitive to SH-reagents (NEM and DTNB) [14,15] and to natural effectors such as nitrosothiols [13,16] and ROS [12].

This cysteine of the ND3 subunit (homologous to Ser-46 of NqoA in *Thermus thermophilus*) is located in a hydrophilic interhelical loop that connects transmembrane segments (TMS) 1 and 2 and that forms part of a solvent-inaccessible ubiquinone reaction chamber. The formation of different semiquinone species as intermediates of electron transfer in complex I [17–19] probably occurs in this chamber as a result of sequential reduction of the substrate ubiquinone molecule. It should be noted, that no semiquinone signal has been observed in the D-form of complex I [20].

The function of the A/D transition is currently under discussion [21]. When respiration rate is decreased *in situ*, *e.g.* at limited oxygen concentration, the turnover of complex I becomes limited due to the lack of oxidised ubiquinone. This promotes de-activation of the enzyme in the minute timescale as observed in highly metabolising tissues such as heart or brain and could be a key event in determining the outcome of ischaemia/reperfusion [11–13]. A detailed characterisation of the differences between the two forms would enable better understanding of the role and mechanisms of the A/D transition. Using two homogenous preparations of bovine heart SMP containing either the A- or the D-form of complex I, we identified conformational differences between the two states. We found that Cys-39 is exposed in the D-form of the enzyme even when complex I was a part of a respiratory chain supercomplex. Further, exposed primary amines in complex I were labelled with fluorescent NHS-esters and a difference gel electrophoresis (DIGE) approach was implemented to compare fluorescent maps of the two forms of the enzyme. The NADPH-containing subunit 39 kDa (NDUFA9) [22] and the mitochondrially encoded subunits ND1 (MT-ND1) and ND3 (MT-ND3) were found to be more exposed in the D-form.

## 2 Materials and methods

### 2.1 Materials

All chemicals were purchased from Sigma-Aldrich, Cy5-maleimide, Cy3-NHS ester and Cy5-NHS ester were supplied by Amersham (Amersham CyDye™ monoreactive NHS Esters), digitonin was purchased from Serva and molecular weight ladder was from Fermentas.

### 2.2 Mitochondrial preparation and SMP isolation

Bovine heart SMP were prepared according to standard procedure [9] and stored in liquid nitrogen.

### 2.3 Activity measurements

Oxidation of NADH was determined spectrophotometrically (Varian Cary® 3000) as a decrease in absorption at 340 nm (ε_340nm_ = 6220 M^− 1^·cm^− 1^) with 165 μM NADH in SET medium (0.25 M sucrose, 0.2 mM EDTA, 50 mM Tris–HCl, pH 7.0) or PBS pH 7.0 buffer containing SMP (10–25 μg of protein/ml). Alternatively, complex I NADH:Q_1_ oxidoreductase activity was assessed: 25 μg SMP were diluted in 1 ml PBS pH 7.5 supplemented with 10 μM NADH to convert the enzyme to the A-form. 1 mM KCN, 30 μM Q_1_ and 165 μM NADH were then added to the suspension and the NADH oxidation rate was measured in the linear part of the curve.

The A/D ratio in each sample was estimated essentially as in [12] by measuring the initial rate of NADH oxidation at 340 nm. SMP (25 μg) were either directly added to 1 ml of measuring buffer (SET or PBS pH 8.8 supplemented with 165 μM NADH and 2 mM MgCl_2_) or incubated with 15 μM NADH at pH 7.2 prior to further dilution in measuring buffer. The measured rates correspond respectively to the activity of only the A-form and the total activity of complex I, *i.e.* the activity after NADH-dependent conversion of all complex I to the A-form.

### 2.4 Preparation of A- and D-forms of complex I

To prepare SMP in which complex I is converted to the D-form, SMP were resuspended to 5 mg/ml in SET or PBS buffers pH 8.0 supplemented with 1 mM malonate and 5 mM MgCl_2_ and incubated at 35 °C for 30–60 min under constant shaking. This treatment resulted in almost complete deactivation of complex I. The resulting sample was then divided in two equal parts, one was kept on ice (D-form) and the other was aerobically re-activated in the presence of 400 μM NADH and a NADH regenerating system (0.1 mg/ml alcohol dehydrogenase from *Saccharomyces cerevisiae*, 1% ethanol) for 20 min at room temperature with constant stirring [16]. This treatment resulted in full activation and maintained complex I in the A-form.

### 2.5 Treatment with covalent reagents

The effects of amino-acid specific reagents were evaluated by measuring the NADH:Q_1_ oxidoreductase activity as described above. The effects of these reagents on the D-form were assessed by measuring activity of the treated D-form after NADH-dependent activation.

Sulfhydryl groups were labelled as described previously [14] with minor modifications. Pelleting was performed by centrifugation at 20,000 *g* at 4 °C for 30 min. SMP were diluted to 5 mg/ml in SET buffer pH 7.5 and complex I was activated in the presence of an NADH-regenerating system. SMP containing the A-form of complex I were then incubated with 30 mM NEM at 15 °C for 15 min. After thiol blockage the reaction was terminated by the addition of 3 volumes of SET buffer pH 7.5 supplemented with 35 mM cysteine. SMP were washed twice in SET buffer pH 7.5. The sample was split into two portions prior to the penultimate centrifugation step. One portion was kept on ice (NEM-treated A-form) and the other one was de-activated (NEM-treated D-form). Both samples were resuspended at 5 mg/ml in SET buffer pH 7.5 and treated with 2 μM Cy5-maleimide at 15 °C for 15 min in the dark. Labelled SMP were then collected by centrifugation and samples were immediately prepared for a BN-PAGE analysis.

For tyrosine labelling, SMP containing A- or D-forms of complex I were diluted to 5 mg/ml and treated with 2 to 15 μM of tetranitromethane (TNM) in SET buffer pH 8.0 prior to activity measurements or incubated with 3 mM *N*-acetylimidazole (NAI) in PBS pH 7.2. For NAI, the reaction was conducted at pH 7.2, which maintains a good balance between the rate of the reaction and the stability of the *O*-acetyltyrosine produced [23]. Reversibility of both reactions was assessed by incubation with 1 mM DTT after treatment with 15 μM TNM or with 100 mM of hydroxylamine after a 1 hour incubation with 3 mM NAI.

For lysine labelling, SMP containing A- or D-forms of complex I were diluted in PBS pH 7.4 [24] to 10 mg/ml and incubated with 10 μM Cy5-NHS esters for 1 h at 10 °C. The reaction was terminated by 10 minute incubation on ice with 50 mM Tris–HCl pH 7.0. Labelled proteins were then immediately prepared for BN-PAGE analysis.

For activity measurements, SMP containing A- or D-forms of complex I were diluted to 1 mg/ml in PBS pH 7.4 and incubated with 8 μM of fluorescein-NHS (F-NHS) for 1 h at 10 °C in the dark. Aliquots were taken every 10 min to measure the NADH:Q_1_ oxidoreductase activity.

### 2.6 Cy3-NHS and Cy5-NHS esters labelling (DIGE)

SMP containing A- or D-forms of complex I were diluted to 1 mg/ml in PBS pH 7.4 and incubated for 1 h at 10 °C in the presence of 8 μM of either Cy3-NHS ester or Cy5-NHS ester. The reaction was stopped by a 10 minute incubation on ice with 50 mM Tris–HCl pH 7.0. The suspension was then centrifuged at 20,000 *g* for 20 min at 4 °C and pellets were prepared for BN-PAGE analysis. Proteins were extracted with 2.5 g of DDM per gram of protein and Cy3/Cy5-labelled samples mixed to a 1:1 ratio (wt/wt). Complex I was isolated on a 4–13% BN-PAGE and differentially labelled subunits from both A- and D-forms were separated with double SDS-PAGE (dSDS-PAGE).

### 2.7 BN-PAGE/dSDS-PAGE analysis

The SMP pellet was processed according to [25] using DDM or digitonin at a ratio of 2.5 g or 6 g of detergent per gram of protein, respectively. Solubilised SMP (150–300 μg of protein) were applied on a 4–13% gradient BN-PAGE. For hrCN-PAGE, a cathode buffer (50 mM tricine, 7.5 mM imidazole, pH 7) supplemented with 0.01% DDM and 0.05% deoxycholate was used [26]. The complex I band was excised from BN-PAGE and dSDS-PAGE (10% SDS-PAGE supplemented with 6 M urea for the first dimension, 16% SDS-PAGE for the second) was performed [14,27]. Gels were then scanned for fluorescence with λ_ex_/λ_em_ 635/670 nm for Cy5-NHS and 532/580 nm for Cy3-NHS using a fluorescence image scanner (FLA-7000, Fujifilm Life Science) and silver-stained as described previously [28].

### 2.8 Fluorescence analysis

Fluorescent scans were analysed with AIDA Image Analyzer (Raytest). For each set of experiments, the intensity of each spot was automatically quantified by the software and the background was subtracted from the signal for each spot. The intensity of fluorescence was then expressed as a percentage of the total fluorescence of all protein spots in the gel. This last step is a prerequisite for comparison since the dyes used do not have the same quantum yield (0.28 for Cy5 and 0.15 for Cy3). A- and D-form samples were both treated with Cy3 and Cy5-NHS esters and measures of all four signals were taken into account to overcome dye-dependent effects for quantification.

### 2.9 Mass spectrometry analysis

Gel spots from CyDye NHS treated A- and D-forms were excised and cut into 1 mm cubes. These were then subjected to in-gel digestion, using a ProGest Investigator in-gel digestion robot (Genomic Solutions, Ann Arbor, MI) using standard protocols [29]. Briefly the gel cubes were destained by washing with acetonitrile and subjected to reduction and alkylation before digestion with trypsin at 37 °C. The supernatant was collected and then the gel pieces extracted twice with 50% acetonitrile, 5% formic acid and then twice with 95% acetonitrile, 5% formic acid and the combined extracts concentrated to 20 μl using a SpeedVac (ThermoSavant).

The peptides were then separated using a nanoLC Ultra 2D plus loading pump and nanoLC as-2 autosampler equipped with a nanoflex cHiPLC chip based chromatography system (Eskigent), using a ChromXP C18-CL trap and column. The peptides were eluted with a gradient of increasing acetonitrile, containing 0.1% formic (5–40% acetonitrile in 5 min, 40–95% in a further 1 min, followed by 95% acetonitrile to clean the column, before reequilibration to 5% acetonitrile). The eluent was sprayed into a TripleTOF 5600 electrospray tandem mass spectrometer (ABSciex, Foster City, CA) and analysed in Information Dependent Acquisition (IDA) mode, performing 250 ms of MS followed by 100 ms MSMS analyses on the 20 most intense peaks seen by MS. The MS/MS data file generated was analysed using the ProteinPilot 4.5 Paragon algorithm (ABSciex) against the Swiss-Prot database Jan 2013 with no species restriction, trypsin as the cleavage enzyme and carbamidomethyl modification of cysteines.

TNM treated active/de-active forms of complex I were isolated by BN-PAGE and subunits were separated by dSDS-PAGE. Spots containing the ND3 subunit were treated with DTT and cysteines were carbamidomethylated with iodoacetamide. This was followed by in-gel digestion with trypsin. Tryptic peptides were subjected to LC–MS/MS analysis on an LTQ-Orbitrap XL (Thermo) mass spectrometer coupled to a nano-HPLC (Agilent). Briefly, peptides were separated in 30 min on an in-house C18 nano-flow column with an acetonitrile gradient (5–45%) containing 0.1% formic acid. This was followed by cleaning with 95% acetonitrile and reequilibration with 5% acetonitrile (15 min each step). Collision induced dissociation (CID) spectra were collected and evaluated by Mascot database searches against UniProt database of *Bos taurus* (26,536 sequences January 2012) with optional modifications on cysteine by carbamidomethylation, mono-, di- and tri-oxidations, modification by acrylamide, mono-oxidation on methionine and nitration of tyrosine.

Label free quantification was conducted using Proteome Discoverer 1.3 Precursor Ions Area Detector node (Thermo Fisher scientific).

## 3 Results

### 3.1 The supercomplex profile after separation by native electrophoresis is not affected by de-activation of complex I

Complex I is found to be a member of several supercomplexes as revealed by native electrophoresis after solubilisation of mitochondrial membranes with digitonin; a mild detergent known to preserve oligomeric association between membrane proteins [25]. Several different higher molecular weight forms can be observed after separation on a gradient native gel [26]. In such supercomplexes, complex I is either associated with complex IV (I + IV), with two copies of complex III (I + III_2_), two copies of complex III and several copies of complex IV (I + III_2_ + IV_x_) [26].

To date, mitochondrial complex I is the only enzyme of the mitochondrial respiratory chain known to undergo a so called A/D conformational change. Whether the association of this enzyme with other complexes is influenced by its conformation remains unclear.

**Fig. 1:**
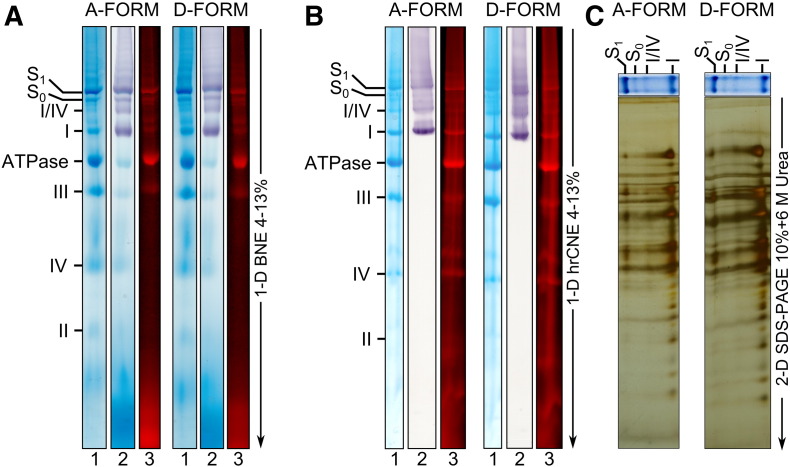
Effect of deactivation of complex I on the supercomplex profile of bovine heart mitochondrial membranes. SMP containing the A- or the D-form of complex I were treated with Cy5-NHS ester as described in Materials and methods. Membranes were solubilised with digitonin and 150 μg of protein for each sample were applied on a 4–13% BN-PAGE (A) or hrCN-PAGE (B). Proteins were visualised by Coomassie Blue staining (lane 1), complex I in-gel activity staining (lane 2) or fluorescent labelling (lane 3). Here and below complexes are labelled according to [26]. I, II, III and IV stand for mitochondrial respiratory complexes I–IV; ATPase for F_1_–F_o_ ATP synthase; I/IV for the association of complexes I and IV. S_0_ for respiratory supercomplex I + III_2_ and S_1_ for I + III_2_ + IV. (C) Silver stained 2D gels (10% SDS-PAGE supplemented with 6 M urea) of supercomplexes S_1_, S_0_, I/IV and complex I isolated from SMP containing the A or D-form of complex I.

The supercomplex profiles of SMP containing A- or D-form complex I were analysed after separation by native electrophoresis. After labelling with a fluorescent lysine-reactive reagent, Cy5-NHS ester, A- and D-forms were solubilised and applied on a 4–13% BN-PAGE (Fig. 1A). No differences in Coomassie staining, complex I in-gel activity or fluorescent signal were detected between the two samples (Fig. 1A, lanes 1 to 3). Complex I from both A- and D-forms migrated at the same level indicating no drastic changes in the molecular weight of A- and D-forms. In-gel activity staining (Fig. 1A, lane 2) revealed four bands corresponding to complex I alone and three types of association with other complexes (I/IV; I + III_2_; I + III_2_ + IV) independent of its conformational state.

Since Coomassie in BN-PAGE quenches fluorescent signals [26,30] samples were also subjected to hrCN-PAGE to test whether supercomplex and subcomplex profiles remained the same for both samples with more sensitive detection (Fig. 1B). Complex I was in-gel activity stained whilst Coomassie staining and fluorescent labelling was used to observe all respiratory chain components. No differences in migration profile were detected. Since a small variation in the composition of these supercomplexes could not easily be detected on BN/CN-PAGE, a second denaturing electrophoresis was run in order to separate the subunits of each entity. As observed after silver staining (Fig. 1C), the composition of the supercomplexes containing A and D forms of complex I was similar.

Taken together these results suggest that the A/D conformational change(s) of complex I has no effect on the association of the enzyme with other respiratory chain complexes *in vitro* as revealed by native electrophoresis. The A/D transition has no effect on the subunit composition of complex I, confirming our previous study [14]. Here we have demonstrated that the A/D transition does not modify the three different supercomplex forms with which complex I is associated.

### 3.2 Cys-39 of the ND3 subunit remains accessible in supercomplexes

SH-reagents such as *N*-ethylmaleimide (NEM) are known to covalently modify Cys-39 of the ND3 subunit in the D-form of the enzyme [14,31]. Once associated in its D-form with other respiratory complexes (*i.e.* complex III and complex IV), this cysteine could be enclosed and inaccessible to modification due to steric hindrance. We tested whether this cysteine is accessible to covalent modification if the D-form of complex I is a constituent of a supercomplex after solubilisation with digitonin.

**Fig. 2:**
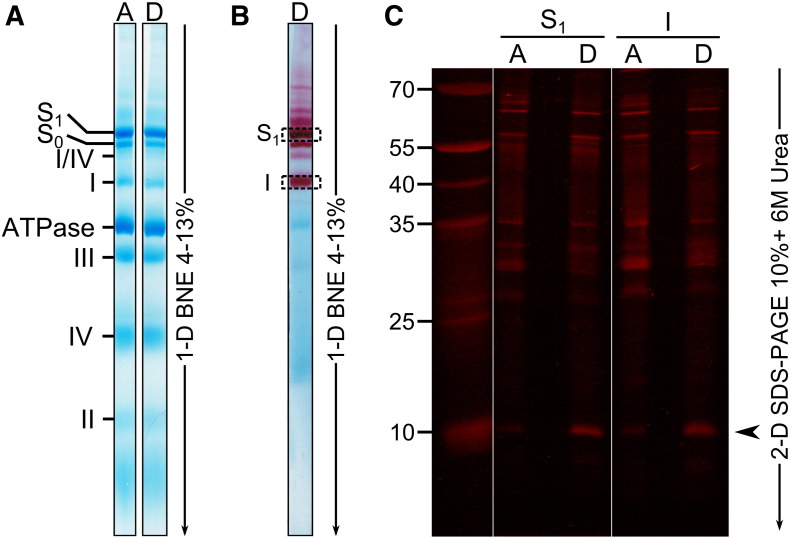
Accessibility of Cys-39 from the ND3 subunit of complex I in the S_1_ supercomplex to chemical modification. A-form of complex I in SMP was treated with NEM. The suspension was divided equally into two parts and one part was de-activated. Both the A and D forms were labelled with NEM and incubated with Cy5-maleimide as described in Materials and methods section. (A) Mitochondrial membrane proteins were solubilised with digitonin and the mitochondrial complexes and supercomplexes (150 μg of protein) were separated by BN-PAGE and stained with Coomassie Blue. (B) In-gel activity staining of complex I was performed and bands S_1_ (complex I as part of supercomplex I + III_2_ + IV) and I (complex I alone) were subjected to a 10% SDS-PAGE + 6 M urea. (C) Representative in gel fluorescence scan after subunit separation on the second denaturing dimension. Approximate molecular weights (kDa) are denoted on the left, the black arrow indicates the ND3 subunit (13.1 kDa).

Using the labelling strategy developed previously [14], the A-form of complex I in SMP was treated with NEM to block available cysteine residues. One part of the sample was then de-activated and both A- and D-forms were treated with fluorescein-NEM in order to modify residues only exposed in the D-form. Proteins were then separated by BN-PAGE, bands corresponding to complex I alone (band I, Fig. 2A and B) and S_1_ supercomplex (I + III_2_ + IV, Fig. 2A and B) were excised and subunits were separated in denaturing conditions. As previously shown [14], Cys-39 of the 13.1 kDa subunit ND3 is the only cysteine residue differentially labelled when complex I was separated by BN-PAGE (Fig. 2C, I). The same fluorescent pattern was observed when complex I was part of the S_1_ supercomplex (Fig. 2A, B). These results suggest that, in the D-form of complex I, Cys-39 of the ND3 subunit is accessible for covalent modification independently of its possible association with complexes III and IV.

### 3.3 The D-form of complex I is highly sensitive to tyrosine reactive reagents

**Fig. A1:**
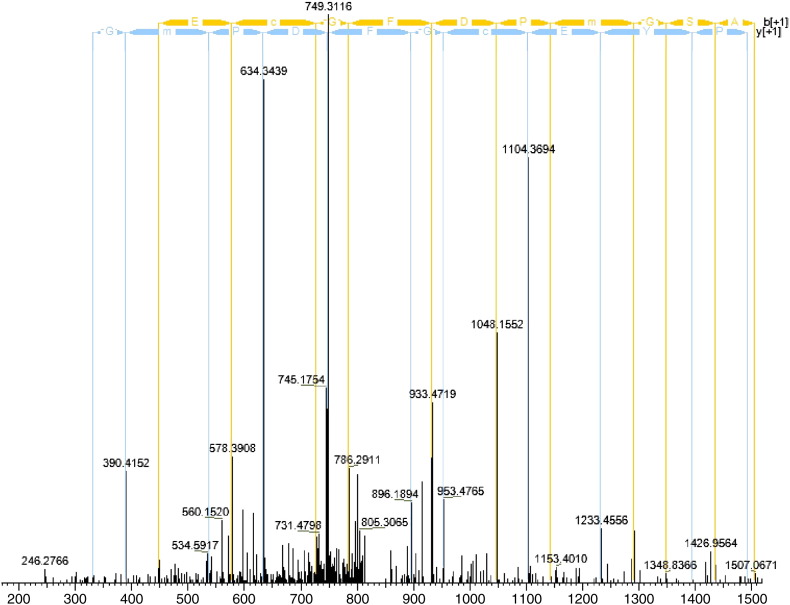
Collision induced dissociation (CID) spectra showing peptide TSPYECGFDPMGSAR of bovine ND3 subunit with trioxidation on cysteine (+ 47.98474 Da), oxidation on methionine (+ 15.99492 Da), charge: + 2, monoisotopic mass m/z: 841.32690 Da, mass deviation from theoretical − 0.24 ppm, Mascot ion score: 61.

**Fig. 3:**
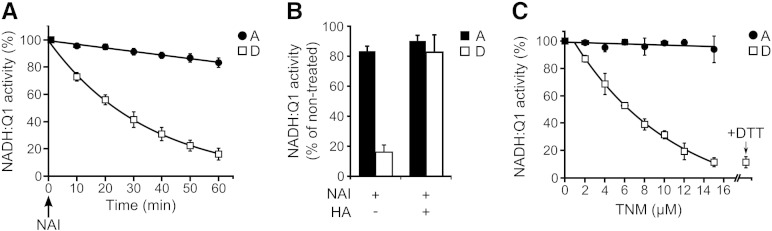
Effect of tyrosine specific reagents on NADH:Q_1_ activity of the A- and D-forms of complex I in SMP. A-form (circle) or D-form (square) of complex I in SMP were treated with NAI and TNM and NADH:Q_1_ reductase activity measured as described in the Materials and methods section. (A) SMP were incubated with NAI and NADH:Q_1_ reductase activity was measured at regular intervals during 60 min; (B) reversibility of NAI modifications was assessed by incubation with hydroxylamine (HA) after treatment with NAI. (C) SMP were treated with increasing concentrations of TNM and activity measured. Reversibility of TNM modification was assessed by incubation with DTT. Data are expressed as a percentage of the activity for non-treated samples. 100% activity corresponds to 1 μmol of NADH·min^− 1^·mg^− 1^ of protein. Data are mean ± S.D. from 3 independent experiments.

We used a different type of chemical modification that specifically targets other amino acids to gain further insight into the structural differences between these two conformations. Tyrosine-specific reagents have been widely used to demonstrate the importance of these residues for a protein's function [32]. Two types of reagent were used to assess any differential accessibility of complex I subunits depending on its conformation. NAI *O*-acetylates protein tyrosyl residues at a pH close to neutral and TNM nitrates these residues in mildly alkaline conditions. As shown in Fig. 3A, the A-form remained active after treatment with NAI, losing only 15% of its activity after a 1 hour incubation, whereas the D to A conversion of the D-form was inhibited by 73%. NAI can also react with other residues, including cysteines *via* sulfhydryl oxidation [32]. Since hydroxylamine deacetylates labile acetyl groups, it was used to assess selective removal of the tyrosine modification. The reducing treatment with hydroxylamine resulted in almost full recovery of the NADH:Q_1_ reductase activity (Fig. 3B). Therefore we concluded that tyrosine modification was the main result of the enzyme treatment with NAI. The modification by NAI is not stable and hydrolysed spontaneously after 4 h (data not shown). In order to determine which modified tyrosine residues could be responsible for the inhibition of the D-form, a stable modification was a prerequisite. The modification of protein tyrosine residues with TNM is known to be stable over a long enough timescale to allow the identification of modified sites by mass spectrometry [33]. As shown in Fig. 3C, TNM treatment of the D-form led to inhibition of its re-activation, whilst the A-form remained fully active even at high TNM concentration. The specificity of the reaction with TNM was assessed by treatment with DTT that would reduce reversibly oxidised cysteines. DTT did not recover TNM inhibition of the D-form, indicating that tyrosine(s) were targeted or cysteines were overoxidised to sulfinic or sulfonic acid. As the inhibition of reactivation of the D-form with TNM appeared to fit the profile of NEM inhibition previously observed [14], ND3 was analysed by mass spectrometry. However a nitration of the tyrosine residue in the identified ND3 peptide (TSPYECGFDPMGSAR) was not detected. Interestingly, Cys-39 in the TNM treated D-form was found to be 14 times more overoxidised compared with the A-form (Table 1 and Fig. A1). Since trioxidation on cysteine is not reduceable by DDT, this thiol modification could prevent the re-activation of complex I in the D-form in the presence of DTT.

The irreversible chemical modification of Cys-39 of the ND3 subunit in the D-form of complex I could then account for the inhibition of the re-activation observed with TNM. However, with the NAI treatment being almost fully reversible, as observed with hydroxylamine, it is unlikely that Cys-39 would be targeted.

A systematic analysis of the 44 subunits of complex I would be necessary to identify the bulk of TNM-modified residues and the differences between the modified A- and D-forms of complex I with mass spectrometry [33] and/or western-blot using antibodies directed towards nitro-tyrosine.

Here, we used a faster method with fluorescent reagent in order to directly identify any subunits involved in conformational changes between the A- and D-forms of complex I.

### 3.4 Lysine specific labelling of A- and D-forms revealed the differential exposure of three subunits

**Fig. 4:**
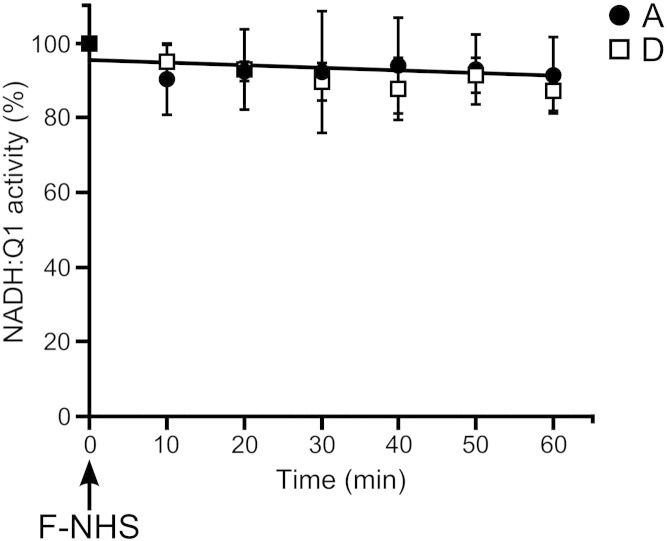
Effect of lysine-specific reagents on the NADH:Q_1_ activity of the A and D forms of complex I in SMP. A-form (circle) or D-form (square) of complex I in SMP was treated with a fluorescent lysine-targeting reagent (F-NHS). Aliquots were taken at regular time intervals and the reaction was terminated by 50 mM Tris–HCl pH 7.0 and NADH:Q_1_ reductase activity measured. 100% corresponds to 1 μmol of NADH·min^− 1^·mg^− 1^ of protein. Data are mean ± S.D. from 3 independent experiments.

In order to identify subunits differentially exposed in the A-form and the D-form we used lysine-specific *N*-hydroxysuccinimide ester based fluorescent dyes. NHS is known to acylate the primary amino-group leading to a covalent modification of the ε-amino-group of lysine or the N-terminal amino-group [24,34]. Unlike cysteine, lysine residues are relatively abundant in proteins permitting the detection of every subunit of complex I and analysis of their conformation-dependent exposure. Indeed, lysines in the 44 mammalian complex I subunits represent around 5.7% of the total number of amino-acids and ND4L, the smallest mitochondrially encoded subunit, is the only one without lysine. By comparison, the average cysteine content is only 1.3%, with 12 subunits without any cysteine residue in their sequence. The effect of lysine modification was assessed by NADH:Q_1_ activity measurement after F-NHS ester treatment. SMP were incubated with 8 μM of F-NHS ester, a concentration that permits the labelling of proteins in non-saturating conditions. In these conditions, activities of both the A- and D-forms remained unaffected (Fig. 4). Any observed differences in dye incorporation would indicate a differential exposure of particular subunits and not a modification of the enzyme structure induced by the reagent.

**Fig. 5:**
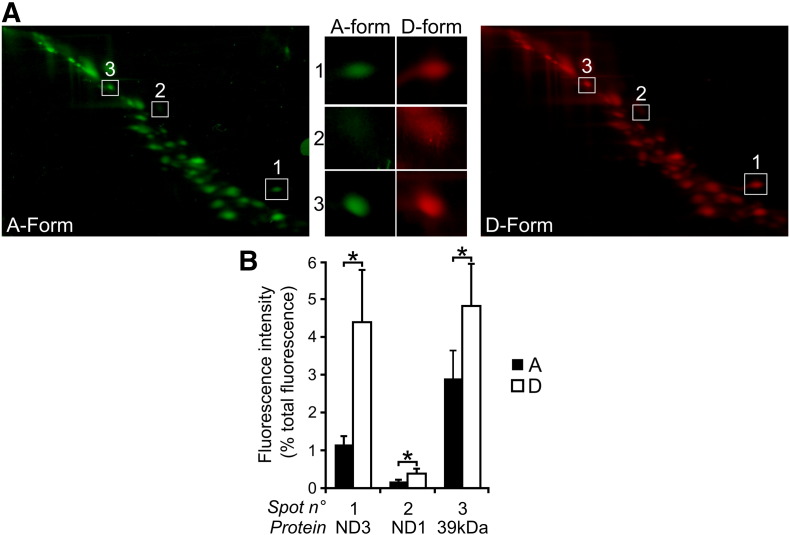
Fluorescent labelling of A- and D-forms of complex I with CyDye™ monoreactive NHS esters. The A- and D-forms of complex I in SMP were respectively treated with Cy3-NHS ester and Cy5-NHS ester and subjected to a DIGE-like approach. (A) Complex I was isolated on a 4–13% polyacrylamide gradient BN-PAGE and subunits from A- and D-forms (right and left panels respectively) were separated by dSDS-PAGE as described in Sections 2.6 and 2.7. Numbers 1, 2 and 3 stand for ND3, ND1 and 39 kDa subunits respectively. (B) Fluorescent intensities were quantified as described in Materials and methods section. The A- and D-forms were both treated with Cy3 and Cy5-NHS esters and measures of all four signals were taken into account for the quantification. Data are mean ± S.D. from at least 5 independent experiments. *p < 0.01 compared to A-form.

A DIGE approach relies on the covalent labelling of two or more samples with a chemical compound bearing the same reactive part and a fluorophore with different spectral properties. After treatment, samples are combined and separated by denaturing electrophoresis. This mixing step circumvents the problem of variability between gels. The A- and D-forms of complex I in SMP were labelled either with cyanine fluorescent Cy3-NHS or Cy5-NHS esters respectively and pooled. Subunits were then separated by dSDS-PAGE (Fig. 5). A reciprocal experiment where A-form was labelled with Cy5-NHS and D-form with Cy3-NHS was also performed. Results of fluorescent intensity quantification with both dyes for each form of complex I (*i.e.* A-Cy3-NHS + D-Cy5-NHS and A-Cy5-NHS + D-Cy3-NHS) are presented in Fig. 5B. Three subunits identified by mass spectrometry (Table A1) were found to be differentially labelled and more exposed in the D-form of complex I. ND3 subunit signal is 4 fold higher in the D-form than in the A-form of complex I. This observation was consistent with earlier reports that showed exposure of Cys-39 of this subunit in the D-form only [14] and validated our approach. Two other subunits were found differentially exposed depending on the conformation of the enzyme. Indeed, 39 kDa (NDUFA9) and ND1 were found to be 1.7 and 2 fold more labelled in the D-form, respectively (Fig. 5B). The 39 kDa subunit is an accessory subunit of mammalian complex I, whereas ND1 is a mitochondrially encoded core subunit involved in proton translocation [4].

For the first time, we demonstrate that ND3 is not the only subunit to become exposed upon deactivation of mitochondrial complex I. A second membrane subunit, ND1 and the accessory subunit 39 kDa are shown to be associated with the structural reorganisation leading to the D-form of the enzyme.

Protein labelling with NHS esters leads to the elimination of tryptic cleavage sites [35] as trypsin mainly cleaves at the carboxyl side of lysine and arginine residues [36]. Membrane proteins, such as ND3 and ND1, are not easily detected by mass spectrometry, preventing us from estimating the region(s) differentially exposed due to a low number of detectable peptides (Table A1). The 39 kDa subunit, thought to have only one TMS [37], was the main candidate for comparison of tryptic peptide profiles from the A- and D-forms of complex I. The higher the number of peptides detected the higher the probability of detecting missed cleavage sites to confirm a different degree of labelling between A- and D-forms.

**Fig. 6:**
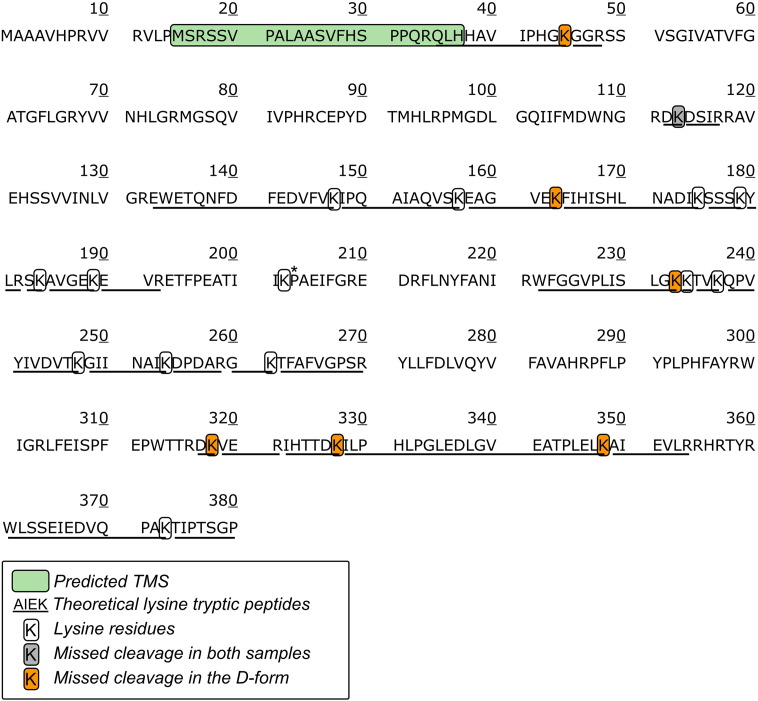
Primary sequence of the bovine 39 kDa subunit (NDUFA9). The hydrophobic segment predicted to be a transmembrane segment [39] is delineated in green. The theoretical tryptic peptides resulting from a cleavage after lysine residues are underlined. The lysine residues are shown with boxes with missed lysine cleavage for both A- and D-form and missed cleavage for the D-form only are indicated by grey boxes and orange boxes respectively. *Non cleavable lysine as followed by a proline residue.

This subunit has 44 theoretical tryptic peptides (PeptideMass Expasy, SwissProt accession number P34943). Among them, 28 are generated by a cleavage after lysine residues (Fig. 6). Lys 113 is the only lysine residue for which no peptides were matched to 39 kDa in both A- and D-forms (Fig. 6). Six missed cleavage sites of 39 kDa from the D-form of complex I were reported (Fig. 6). These residues were all observed/cleaved in the A-form. It is therefore conceivable that the difference in the fluorescent intensity between A- and D-forms of complex I for the 39 kDa subunit corresponds to a modification at Lys-45, 163, 233, 318, 327 and 348.

## 4 Discussion

An intriguing feature of mitochondrial complex I is its ability to switch from an active (A) form to a de-active (D) form. The role of this transition in enzyme regulation and functioning still remains to be characterised in detail. Despite recent progress detailing the existence of two forms of mitochondrial complex I *in situ* [10–13] and the important role played by the A/D transition in mitochondrial response to hypoxia [12,13,16,38], little is known about the conformational differences between the two forms.

The A to D transition of the enzyme in bovine SMP is characterised by high activation energy [9], suggesting significant conformational changes in complex I upon deactivation. Until now, the only part of complex I known to be differentially exposed depending on the A/D conformation is the matrix-facing loop connecting the first and second TMS of the ND3 subunit. Indeed, ND3's single cysteine (Cys-39, equivalent to Ser-46 of Nqo7 in *T. thermophilus*) is exposed to the matrix side in the D-form only, where it is susceptible to covalent modification by SH-reagents, nitric oxide metabolites and ROS [12,14,16,31].

In the present study, we addressed two different questions to gain additional understanding of the A/D transition: (i) Does the de-activation of complex I affect the supercomplex assembly? (ii) Apart from ND3, are there any additional subunits associated with conformational changes upon de-activation?

### 4.1 The D-form of complex I does not affect supercomplex formation *in vitro*

Complex I has been proposed as an ‘enhancer’ of supercomplex formation [39–41], suggesting a central role in their assembly/regulation. It has recently been suggested that MCJ, a methylation-controlled J protein belonging to the DnaJC subfamily of co-chaperones, could interact with and negatively regulate mitochondrial complex I. The presence of this protein would decrease both the formation of supercomplexes and complex I activity; thus MCJ may play a role in the regulation of the A/D transition [42]. However, to our knowledge, the influence of complex I conformation on the degree of association with other members of the respiratory chain has never been examined.

In order to investigate this, the supercomplex profile of SMP containing the A- or D-forms of complex I were analysed after separation by two types of native electrophoresis. We did not find any differences in the migration profile and content of supercomplexes depending on the conformation of complex I. Our data suggests that the conformation of complex I does not affect the structural formation or stability of supercomplexes in bovine heart SMP. Indeed, the fact that no extra bands could be detected with in-gel activity staining or highly sensitive fluorescent labelling suggests that complex I can adopt two different conformations whilst being part of a higher molecular weight complex. It is important to note that, unlike NADH:ubiquinone reductase, the commonly used rotenone-insensitive NADH:tetrazolium reaction for in-gel activity measurements is catalysed by both forms of the enzyme with the same rate.

Therefore, the A/D transition would correspond to changes in the enzyme structure rather than changes of the macromolecular organisation within supercomplexes.

The D-form of complex I is characterised by the exposure of Cys-39 of the ND3 subunit. Complex I is known to associate with complexes III and IV. In the S_1_ supercomplex, the complex III dimer has been shown to locate in the arc of the bent membrane part of complex I, allowing the ubiquinone binding sites of both complexes I and III to face each other [43]. In the D-form, Cys-39 of the ND3 subunit (located in the vicinity of the ubiquinone binding site [4]) could be masked and inaccessible to chemical modification once associated with respiratory complexes III and IV. To test this hypothesis, complex I was modified with NEM/F-NEM as described in [14]. As expected, we observed fluorescent labelling of ND3 in the separated complex I band. This labelling was preserved even when complex I was part of the I + III_2_ + IV supercomplex. Our result indicates that the potential interaction between complex I and complex III in the mitochondrial membrane does not significantly affect the accessibility of Cys-39 in the D-form of the enzyme.

### 4.2 Specific amino-acid labelling unveils the association of ND1 and 39 kDa subunits with structural reorganisation upon de-activation

To characterise the other potential differences in the accessibility of complex I subunits, we decided to test several amino acid specific covalent reagents. A- and D-forms of complex I in bovine heart SMP were treated with tyrosine-specific reagents, NAI and TNM. These compounds covalently modify tyrosine residues with a high level of specificity. We found that both reagents inhibited the re-activation of the D-form of the enzyme, suggesting other amino acids are exposed in the D-form. Modification of two tyrosines (Tyr-30 and Tyr-37) in close proximity to Cys-39 of the ND3 subunit may account for the sensitivity of the D-form to NAI and TNM. A mass spectrometric analysis of the TNM-modified ND3 subunit did not permit us to locate the modification site(s) on tyrosine residues [14,44]. However, the differential exposure of tyrosine residues from other subunits during the A to D transition cannot be excluded and is currently under investigation. Besides tyrosine nitration it has been reported that treatment with TNM alternatively oxidises cysteine. To enhance the specificity of TNM treatment for tyrosine nitration NEM can be added to the labelling mixture [45]. Because cysteine labelling irreversibly alters the enzyme, ‘locking’ de-activated complex I in the D-form, it had to be omitted in order to observe the effects of TNM only.

This enabled us to identify sulfonic acid modification on Cys-39 after TNM treatment resulting in an irreversible de-activation of complex I. Since sulfonic acid modification could occur *in vivo* upon oxidative stress [46] complex I could be arrested in the D-form resulting in complex I deficiency.

We used lysine specific fluorescent NHS-esters combined with a DIGE-like approach to gain further insight into the structural differences between these two conformations. We identified three subunits that were unequivocally more exposed in the D-form: ND3, ND1 and 39 kDa (NDUFA9).

The differential exposure of the ND3 subunit confirmed our previous results [14] and validated our approach. The bovine ND3 subunit contains three lysine residues, all of which are potentially accessible from the matrix side. Lys-33 and Lys-54 belong to the Cys-39 containing hydrophilic loop whereas Lys-109 is located in the soluble C-terminus. It is thus tempting to speculate that Lys-109 is labelled in both forms whereas Lys-33 and Lys-54 are labelled only in the D-form, accounting for the difference in the intensity of fluorescence. Taken together these data may suggest that a significant region of the ND3 subunit (probably between Trp-22 and Lys-54) becomes exposed when complex I undergoes deactivation.

The 39 kDa (NDUFA9) nuclear encoded accessory subunit was found to be associated with structural rearrangement during the A/D transition and therefore more accessible for chemical modification upon deactivation of complex I. A previous study performed in our laboratory [44] revealed that ND3 can be crosslinked with 39 kDa subunit by a heterobifunctional —NH_2_/—SH specific 6.8 Å crosslinker only when complex I is in the D-form. This observation could be accounted for by the individual movement of the hydrophilic loop of ND3 towards 39 kDa subunit or by a combined rearrangement of the two subunits. The work presented here strongly suggests that 39 kDa subunit undergoes concerted structural rearrangement along with ND3 during de-activation. The 39 kDa subunit is thought to be anchored to the membrane part of the enzyme *via* a single hydrophobic transmembrane helix [37] and positioned at the crucial junction region [47] between the membrane and the hydrophilic matrix domain of complex I [2,47,48]. This corresponds to the location of the NqoA subunit (homologous to ND3) in the *T. thermophilus* enzyme structure [4,49].

The 39 kDa (NDUFA9) subunit belongs to the family of short-chain dehydrogenase/reductases. It contains a nucleotide binding Rossmann fold motif [50] where one molecule of NADPH is non-covalently bound [22]. This bound form of NADPH is not involved in catalytic turnover and is present in the reduced form [51,52].

Mutations affecting the NADPH binding site in *Yarrowia lipolytica* protein have only a minor effect on the ubiquinone reductase activity of complex I, suggesting a stabilising function for this nucleotide rather than a role in biosynthesis of complex I [51]. However, disruption of this subunit in HEK cells [53,54] or mutation of a highly conserved arginine [55] results in defective complex I assembly or neonatal fatality respectively. Taken together, this indicates the importance of the NDUFA9 subunit for complex I functioning and regulation.

Another main finding of this study is that ND1, a core membrane subunit, is also affected by structural rearrangements upon deactivation of complex I. The recently obtained three-dimensional structure of the bacterial enzyme clearly revealed the quinone reaction chamber [4]. This cavity is formed by four different subunits. The ND1 and ND3 subunits (Nqo8 and Nqo7 in *T. thermophilus* complex I) form the cavity at the quinone entrance side, accommodating the hydrophobic isoprenoid tail. The 49 kDa and PSST subunits (Nqo4 and Nqo6 in *T. thermophilus* complex I) accommodate the quinone head group from the N2 terminal cluster side [4]. The entry point of this cavity is delineated by TMS1, TMS6 and AH1 of ND1, as well as TMS1 from ND3. ND1 and ND3 narrow the entrance of the cavity, so that when quinone binds, the cavity would be enclosed and separated from the bulk solvent. The bovine ND1 subunit has a unique cysteine (Cys-301) located in TMS8 and facing the matrix side. Fluorescein-NEM treatment of both the A- and the D-form of complex I resulted in equal fluorescent labelling of this subunit [14], suggesting a similar exposure of TMS8 located at the C-terminal extremity. Among seven lysines found in the bovine ND1 sequence, two are located in the vicinity of TMS1 and AH1 of ND1. Based on the *T. thermophilus* structure, three other ND1 lysine residues (Lys-54, Lys-58 and Lys-126) are located within 10 Å of the hydrophilic loop of ND3 [4]. Their exposure could be modified upon de-activation, and structural rearrangements within this region could potentially account for the differential labelling observed for ND1.

Mutations in human *MT-ND1* are associated with the appearance of several severe syndromes, including LHON and MELAS. To date, 41 disease-associated mutations on *MT-ND1* have been reported [56]. Most of these mutations are located in the hydrophilic loop of ND1 (for review, see [57]). Studies on the molecular consequences of such mutations have often been conducted in bacterial models of complex I [58–61]. These studies showed varied consequences at the level of complex I, ranging from an assembly defect to a decrease in the enzyme activity. A difference in the sensitivity to inhibitors was also demonstrated, suggesting an alteration of the quinone binding pocket [59,61]. Pathological mutations in *MT-ND1* leading to impairment in complex I activity (but not assembly) could also suggest a defect in the regulation of the A/D balance. The involvement of ND1 (and ND3) on the A/D transition demonstrated in this study raises questions as to the use of bacterial models in the study of human point mutations found on *MT-ND1* and *MT-ND3*. Since the bacterial complex I does not display the A/D transition this may be misleading for the study of the overall functional consequences of such mutations, not at the level of the enzyme as such, but for global consequences on the respiratory chain.

### 4.3 Potential mechanisms leading to the de-activation of mitochondrial complex I

Deactivation results in rearrangements in ND3 as well as in ND1 subunits in the region of the quinone binding pocket and potentially accounts for the labelling of ND3 (by SH-reagents and NHS esters) and ND1 (by NHS esters) observed in the D-form only. It is possible that deactivation of mitochondrial complex I leads to the disruption of the enclosed quinone binding chamber due to a concerted structural rearrangement of these two crucial membrane subunits. The processes of reduction of the physiological ubiquinone molecule and transmission of the redox energy into putative proton translocating channel(s) could then be disrupted. Interestingly, 49 kDa and PSST subunits were equally labelled in both forms of complex I, suggesting rather selective conformational change(s) in the membrane part of the enzyme at the level of the entrance of the quinone binding cavity. It is tempting to speculate that the initial slow turnover (k_cat_ ~ 1–10 min^− 1^) necessary for activation of the D-form corresponds in fact to the formation of a sealed chamber able to accommodate ubiquinone.

An alternative explanation is that a rearrangement of ND3 and ND1 subunits during D to A transition is required for formation of a mechanistic coupling link between the quinone reduction step and conformationally driven proton translocation *via* antiporter-like channels in the membrane. However, this is less likely since the deactivation results in almost full inhibition of NADH:ubiquinone reductase activity of the D-form.

Interestingly, it has been found that the addition of rotenone to a mixture of A- and D-forms in SMP (i) partially protects the A-form against de-activation and (ii) partially converts the D-form to an A-like conformation. This was observed by measurement of the reverse electron transfer activity after removal of rotenone with BSA. Rotenone could act (i) as a clamp for the A-form of the enzyme and (ii) induce conformational changes in the D-form similar to the transitory state so that the enzyme-inhibitor complex can undergo activation without a turnover [62].

Therefore, the rate of activation would be limited by the time required for rearrangements of the enzyme structure, *i.e.* formation of the pathway/binding site for the ubiquinone molecule and/or reduction of quinone at the terminal cluster N2.

## 5 Conclusions

Our results are in line with the known plasticity of the junction region (Q-module) between the hydrophilic module that operates the electron transfer from NADH to the terminal N2 FeS cluster (N-module) and the proton pumping module (P-module) [63]. Indeed, the formation of a subcomplex Iδ in the *Y. lipolytica* enzyme after treatment with lauryl dimethylamine oxide showed that the hydrophobic subunits of this connecting region (including ND1 and ND3) are the first ones to be dissociated [64,65], suggesting a difference in stability. The flexibility of this region would then not only be crucial for energy transduction, as previously suggested [66–68], but also for the mechanism of conformational change during A/D transition, as discussed in the present paper. These two processes may however involve different types of structural rearrangement as it is unlikely that the A-form of complex I undergoes extensive conformational change during steady-state catalytic turnover [4]. The activation/de-activation process would then probably involve a higher degree of structural reorganisation compared to that required for energy transduction during catalysis. The nature of the driving force behind concerted conformational changes of at least three subunits in the A/D transition (ND3, ND1 and 39 kDa) remains to be answered.

## Figures and Tables

**Fig. A2:**
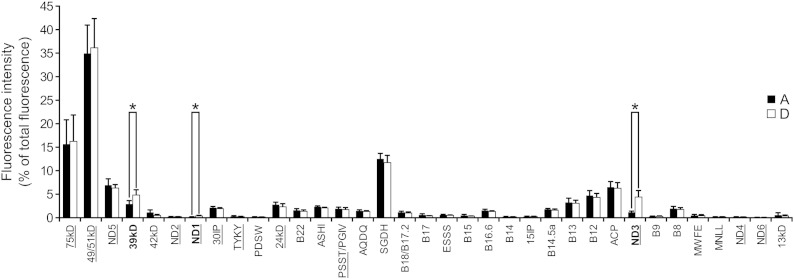
Normalised fluorescence intensities for every detectable subunit of complex I in A and D-forms after labelling with NHS-esters. Fluorescence intensities are presented as percentage of the total fluorescence as described. SMP containing the A-form (black bars) and D-form (grey bars) of complex I were treated with both Cy3 and Cy5-NHS esters to overcome dye-dependent effects.

**Table 1:** Label free quantification of the modified ND3 peptide.

TSPYECGFDPMGSAR^a^	A-form^b^	D-form^b^	Ratio D/A	Mascot score	
				A	D
C6(carbamidomethyl)	1149961	1590834	1.4	33	65
C6(carbamidomethyl); M11(oxidation)		633153			30
C6(propionamide)	4725446			60	
C6(trioxidation)	969553	14305278	14.8	34	46
C6(trioxidation); M11(oxidation)		13997917			61
C6(carbamidomethyl + propionamide)	5875408	1590834	0.3		
C6(trioxidation)	969553	14305278	14.8		

The two last rows summarise the obtained results for carbamidomethyl + propionamide and trioxidation modifications of Cys-39.

a Modifications found on the unique analysed peptide.

b Peptide areas extracted from the HPLC chromatograms with the precursor ions area detector node implemented into the Proteome Discoverer workflow.
