# Supplementary material for: ND3, ND1 and 39 kDa subunits are more exposed in the de-active form of bovine mitochondrial complex I
Source: Biochim Biophys Acta. 2014 Jun;1837(6):929–39. doi: 10.1016/j.bbabio.2014.02.013 (PMC4331043; doi:10.1016/j.bbabio.2014.02.013)
Supplement: Supplementary file 1 — Supplementary material. [file mmc1.doc]

**Table A1**

Peptide identification after tryptic digestion of spots 1-3 presented in Figure 5.

| **Spot^a^** | **Confidence** | **Sequence** | **Modifications** | **Cleavages** | **Δmass,**  **Da** | **Precursor**  **MW, Da** | **Precursor**  **m/z** | **Charge**  **State, z** |
| --- | --- | --- | --- | --- | --- | --- | --- | --- |
| 1^A^ | 99 | TSPYECGFDPMGSAR | Carbamidomethyl(C)@6;Oxidation(M)@11 |  | 0.0009 | 1689.6772 | 845.8459 | 2 |
| 2^A^ | 99 | VLGYMQLR | Oxidation(M)@5 |  | 0.0006 | 994.5276 | 498.2711 | 2 |
| 2^D^ | 99 | KVLGYMQLR | Oxidation(M)@6 | missed K-V@1 | -0.0006 | 1122.6212 | 562.3179 | 2 |
|  | 99 | VLGYMQLR |  |  | 0.0014 | 978.5334 | 490.274 | 2 |
|  | 99 | YALIGALR |  |  | -0.0015 | 875.5215 | 438.768 | 2 |
|  | 98.8 | VLGYMQLR | Oxidation(M)@5 |  | -0.0019 | 994.5251 | 498.2698 | 2 |
| 3^A^ | 99 | AVEHSSVVIN |  | cleaved N-L@C-term | 0.0021 | 1053.5477 | 527.7811 | 2 |
|  | 99 | AVEHSSVVINLVGR |  |  | -0.0007 | 1478.8198 | 493.9472 | 3 |
|  | 99 | AVEHSSVVINLVGREWE | Amidated@C-term | cleaved E-T@C-term; missed R-E@14 | -0.0052 | 1921.9956 | 641.6726 | 3 |
|  | 99 | AVGEKEVR | Formyl(K)@5 | missed K-E@5 | 0.0014 | 914.4837 | 458.2491 | 2 |
|  | 99 | DVTKGIINAIKDPDAR |  | cleaved V-D@N-term; missed K-G@4; missed K-D@11 | -0.0049 | 1724.9374 | 432.2416 | 4 |
|  | 99 | EAGVEKFIHISHLNADIK |  | missed K-F@6 | 0.0011 | 2020.0753 | 506.0261 | 4 |
|  | 95.4 | EAGVEKFIHISHLNADIK | Formyl(K)@6 | missed K-F@6 | -9.47E-5 | 2048.069 | 513.0245 | 4 |
|  | 99 | EDRFLNYFANIR |  | missed R-F@3 | 0.0011 | 1556.7748 | 519.9322 | 3 |
|  | 99 | ETFPEATIIKPA |  | cleaved A-E@C-term | -0.0026 | 1315.6998 | 658.8572 | 2 |
|  | 99 | ETFPEATIIKPAEIFGR |  |  | 0.0051 | 1918.0248 | 640.349 | 3 |
|  | 99 | ETFPEATIIKPAEIFGR | Cation:Na(E)@5 |  | 0.0026 | 1940.0046 | 647.6755 | 3 |
|  | 99 | ETFPEATIIKPAEIFGR | Formyl(K)@10 |  | 0.0021 | 1946.0171 | 649.6796 | 3 |
|  | 99 | ETFPEATIIKPAEIFGR | Carbamidomethyl(K)@10 |  | 0.0067 | 1975.0482 | 659.3567 | 3 |
|  | 95.4 | ETFPEATIIKPAEIFGR | Phospho(T)@2 |  | 0.0003 | 1997.9874 | 1000.0004 | 2 |
|  | 99 | ETFPEATIIKPAEIFGREDR | Carbamidomethyl(K)@10 | missed R-E@17 | -0.0014 | 2375.2104 | 594.8099 | 4 |
|  | 99 | ETFPEATIIKPAEIFGREDR | Formyl(K)@10 | missed R-E@17 | 0.0035 | 2346.1892 | 783.0703 | 3 |
|  | 99 | ETFPEATIIKPAEIFGREDR | Cation:Na(E)@5 | missed R-E@17 | -0.0025 | 2340.1702 | 781.064 | 3 |
|  | 95.4 | ETFPEATIIKPAEIFGREDR | Phospho(T)@2 | missed R-E@17 | -0.0075 | 2398.1494 | 800.3904 | 3 |
|  | 99 | EWETQNFDFEDVFVK |  |  | 0.0026 | 1931.8602 | 966.9374 | 2 |
|  | 99 | FIHISHLNADIK |  |  | -0.002 | 1406.7651 | 469.9289 | 3 |
|  | 99 | FIHISHLNADIK | Carbamidomethyl(S)@5 |  | -0.0034 | 1463.7853 | 488.9357 | 3 |
|  | 99 | FIHISHLNADIKSSSK |  | missed K-S@12 | -0.0022 | 1795.9557 | 449.9962 | 4 |
|  | 99 | FIHISHLNADIKSSSK | Carbamidomethyl(K)@12 | missed K-S@12 | -0.0015 | 1852.9781 | 464.2518 | 4 |
|  | 99 | FIHISHLNADIKSSSK | Formyl(K)@12 | missed K-S@12 | -0.0004 | 1823.9528 | 608.9915 | 3 |
|  | 99 | FLNYFANIR |  |  | 0.0006 | 1156.6035 | 579.309 | 2 |
|  | 98.7 | FLNYFANIR | Trioxidation(Y)@4 |  | 0.0003 | 1204.588 | 603.3012 | 2 |
|  | 99 | GIINAIKDPDAR | Formyl(K)@7 | missed K-D@7 | -0.0028 | 1309.696 | 655.8554 | 2 |
|  | 99 | GIINAIKDPDAR |  | missed K-D@7 | -0.0004 | 1281.7035 | 428.2418 | 3 |
|  | 97.6 | GIINAIKDPDAR | Propionamide(K)@7 | missed K-D@7 | 0 | 1352.7411 | 451.921 | 3 |
|  | 95.4 | GIINAIKDPDAR | Carbamidomethyl(K)@7 | missed K-D@7 | -0.005 | 1338.7207 | 447.2475 | 3 |
|  | 99 | GKTFAFVGPSR | Formyl(K)@2 | missed K-T@2 | -0.0013 | 1193.618 | 597.8163 | 2 |
|  | 99 | GKTFAFVGPSR |  | missed K-T@2 | 0.001 | 1165.6252 | 583.82 | 2 |
|  | 99 | GSQVIVPHR |  | cleaved M-G@N-term | 0.0019 | 991.5583 | 496.7864 | 2 |
|  | 99 | IHTTDKILPHLPGLEDLGVEATPLELK |  | missed K-I@6 | -0.0006 | 2948.6218 | 738.1627 | 4 |
|  | 99 | ILPHLPGLEDLGVEATPLELK |  |  | 0.0026 | 2253.2646 | 752.0955 | 3 |
|  | 99 | INAIKDPDAR |  | cleaved I-I@N-term; missed K-D@5 | -0.0013 | 1111.5973 | 556.8059 | 2 |
|  | 99 | IPQAIAQVSK |  |  | -0.0019 | 1053.6163 | 527.8154 | 2 |
|  | 99 | IPQAIAQVSKEAGVEK |  | missed K-E@10 | -0.0052 | 1666.9203 | 834.4674 | 2 |
|  | 99 | IPQAIAQVSKEAGVEK | Formyl(K)@10 | missed K-E@10 | 0.0027 | 1694.9231 | 848.4688 | 2 |
|  | 95.4 | IPQAIAQVSKEAGVEK | Carbamidomethyl(K)@10 | missed K-E@10 | -0.0033 | 1723.9438 | 575.6551 | 3 |
|  | 99 | KTVKQPVYIVDVTK | Formyl(K)@4 | missed K-T@1; missed K-Q@4 | -0.0002 | 1644.9452 | 549.3222 | 3 |
|  | 99 | KTVKQPVYIVDVTK |  | missed K-T@1; missed K-Q@4 | -0.0003 | 1616.9496 | 539.9905 | 3 |
|  | 99 | KTVKQPVYIVDVTK | GlyGly(K)@4 | missed K-T@1; missed K-Q@4 | 0.002 | 1730.995 | 578.0056 | 3 |
|  | 99 | KTVKQPVYIVDVTK | GlyGly(K)@1 | missed K-T@1; missed K-Q@4 | 0.002 | 1730.995 | 578.0056 | 3 |
|  | 99 | KTVKQPVYIVDVTK | Carbamidomethyl(K)@4; Deamidated(Q)@5 | missed K-T@1; missed K-Q@4 | 0.0021 | 1674.9576 | 559.3265 | 3 |
|  | 99 | LFEISPFEPWTTR | Oxidation(P)@9 |  | 0.0063 | 1637.8153 | 819.9149 | 2 |
|  | 99 | LFEISPFEPWTTR | Cation:Na(E)@3 |  | -0.0014 | 1643.7946 | 548.9388 | 3 |
|  | 99 | LFEISPFEPWTTR |  |  | -0.0004 | 1621.8137 | 811.9141 | 2 |
|  | 99 | LFEISPFEPWTTR | Dioxidation(W)@10 |  | -0.0032 | 1653.8007 | 827.9076 | 2 |
|  | 99 | LFEISPFEPWTTR | Dioxidation(P)@9 |  | -0.0032 | 1653.8007 | 827.9076 | 2 |
|  | 99 | LFEISPFEPWTTR | Phospho(S)@5 |  | -0.0098 | 1701.7706 | 851.8926 | 2 |
|  | 99 | LFEISPFEPWTTRDKVER | Phospho(S)@5 | missed R-D@13; missed K-V@15 | 0.004 | 2329.1187 | 777.3801 | 3 |
|  | 99 | LHHAVIPHGKGGR | Formyl(K)@10 | cleaved Q-L@N-term; missed K-G@10 | 0.0005 | 1405.7695 | 469.5971 | 3 |
|  | 99 | MGSQVIVPH | Oxidation(M)@1 | cleaved H-R@C-term | 0.0015 | 982.4921 | 492.2533 | 2 |
|  | 99 | MGSQVIVPH |  | cleaved H-R@C-term | -0.008 | 966.4877 | 484.2511 | 2 |
|  | 99 | MGSQVIVPHR | Carbamidomethyl(H)@9 |  | -0.0045 | 1179.6136 | 590.8141 | 2 |
|  | 99 | MGSQVIVPHR |  |  | -0.0004 | 1122.5964 | 562.3055 | 2 |
|  | 99 | MGSQVIVPHR | Oxidation(M)@1; Carbamidomethyl(H)@9 |  | 0.0009 | 1195.614 | 598.8143 | 2 |
|  | 99 | MGSQVIVPHR | Oxidation(M)@1; Carbamidomethyl(H)@9 |  | 0.0009 | 1195.614 | 598.8143 | 2 |
|  | 99 | NAIKDPDAR |  | cleaved I-N@N-term; missed K-D@4 | 0.0022 | 998.5168 | 500.2657 | 2 |
|  | 99 | QPVYIVDVTK |  |  | 0.0007 | 1160.6449 | 581.3297 | 2 |
|  | 99 | QPVYIVDVTK | Gln->pyro-Glu@N-term; Cation:Na(D)@7 |  | -0.0014 | 1165.598 | 583.8064 | 2 |
|  | 99 | QPVYIVDVTK | Gln->pyro-Glu@N-term |  | 0.0006 | 1143.618 | 572.8164 | 2 |
|  | 99 | QPVYIVDVTK | Trioxidation(Y)@4 |  | -0.0034 | 1208.6255 | 605.32 | 2 |
|  | 97.6 | QPVYIVDVTK | Cation:Na(D)@7 |  | -0.0002 | 1182.6261 | 592.3202 | 2 |
|  | 99 | RAVEHSSVVINLVGR | Phospho(S)@7 | missed R-A@1 | -0.0076 | 1714.8801 | 572.6341 | 3 |
|  | 99 | RAVEHSSVVINLVGR |  | missed R-A@1 | 0.0013 | 1634.9231 | 545.9816 | 3 |
|  | 95.4 | SSSKYLR | Formyl(K)@4 | missed K-Y@4 | -0.0014 | 867.4437 | 434.7656 | 2 |
|  | 99 | SSVSGIVATVFGATGFLGR |  |  | 0.0039 | 1824.9772 | 609.333 | 3 |
|  | 99 | TFAFVGPSR |  |  | 0.0007 | 980.5087 | 491.2601 | 2 |
|  | 99 | TVKQPVYIVDVTK |  | missed K-Q@3 | -0.0023 | 1488.8529 | 497.2916 | 3 |
|  | 99 | TVKQPVYIVDVTK | Formyl(K)@3 | missed K-Q@3 | -0.0021 | 1516.848 | 759.3751 | 2 |
|  | 99 | TVKQPVYIVDVTK |  | missed K-Q@3 | -0.0023 | 1488.8529 | 497.2916 | 3 |
|  | 99 | TVKQPVYIVDVTK | GlyGly(K)@3 | missed K-Q@3 | 0.0031 | 1602.9012 | 535.3077 | 3 |
|  | 99 | TVKQPVYIVDVTK | Sulfo(Y)@7 | missed K-Q@3 | 0.0005 | 1568.8125 | 785.4135 | 2 |
|  | 99 | TVKQPVYIVDVTK | Carbamidomethyl(K)@3 | missed K-Q@3 | -0.0052 | 1545.8715 | 773.943 | 2 |
|  | 98.7 | TVKQPVYIVDVTK | Phospho(T)@1 | missed K-Q@3 | -0.009 | 1568.8125 | 785.4135 | 2 |
|  | 99 | TYRWLSSEIEDVQPAK | Dioxidation(W)@4 | missed R-W@3 | -0.0024 | 1952.9454 | 651.9891 | 3 |
|  | 99 | TYRWLSSEIEDVQPAK | Oxidation(W)@4 | missed R-W@3 | -0.0048 | 1936.9482 | 646.6567 | 3 |
|  | 99 | TYRWLSSEIEDVQPAK |  | missed R-W@3 | -0.0038 | 1920.9543 | 641.3254 | 3 |
|  | 99 | WFGGVPLISLGK |  |  | 0.0002 | 1272.7233 | 637.3689 | 2 |
|  | 99 | WFGGVPLISLGK | FormaldehydeAdduct(W)@1 |  | 0.0015 | 1284.7247 | 643.3696 | 2 |
|  | 99 | WFGGVPLISLGK | Oxidation(W)@1 |  | 0.0007 | 1288.7186 | 645.3666 | 2 |
|  | 99 | WFGGVPLISLGK | Dioxidation(W)@1 |  | 0.004 | 1304.7169 | 653.3657 | 2 |
|  | 99 | WFGGVPLISLGKK |  | missed K-K@12 | 0.0023 | 1400.8203 | 467.9474 | 3 |
|  | 99 | WFGGVPLISLGKK | Carbamidomethyl(K)@13 | missed K-K@12 | -0.0009 | 1457.8386 | 486.9535 | 3 |
|  | 99 | WFGGVPLISLGKK | Formyl(K)@13 | missed K-K@12 | 0.0011 | 1428.8138 | 715.4143 | 2 |
|  | 99 | WFGGVPLISLGKK | Dioxidation(W)@1 | missed K-K@12 | 0.002 | 1432.8099 | 717.4116 | 3 |
|  | 97.6 | WFGGVPLISLGKK | Propionamide(K)@13 | missed K-K@12 | 0.0023 | 1471.8574 | 491.6264 | 3 |
|  | 99 | WLSSEIEDVQPAK |  |  | -0.0024 | 1500.7437 | 751.3791 | 2 |
|  | 99 | WLSSEIEDVQPAK | Cation:Na(E)@7 |  | -0.003 | 1522.7251 | 762.3698 | 2 |
|  | 99 | WLSSEIEDVQPAK | Dioxidation(W)@1 |  | -0.0031 | 1532.7329 | 767.3737 | 2 |
|  | 99 | WLSSEIEDVQPAK | FormaldehydeAdduct(W)@1 |  | -0.0017 | 1512.7444 | 505.2554 | 3 |
|  | 99 | WLSSEIEDVQPAK | Cation:Na(E)@7 |  | -0.003 | 1522.7251 | 762.3698 | 2 |
|  | 99 | WLSSEIEDVQPAK | Cation:Na(D)@8 |  | -0.003 | 1522.7251 | 762.3698 | 2 |
|  | 99 | WLSSEIEDVQPAK | Oxidation(W)@1 |  | -0.0052 | 1516.7357 | 759.3751 | 2 |
|  | 99 | WLSSEIEDVQPAK | Carbamidomethyl(E)@7 |  | 0.0036 | 1557.7711 | 779.8928 | 2 |
|  | 99 | WLSSEIEDVQPAKTIPTSGP | Phospho(S)@18 | missed K-T@13 | -0.0068 | 2234.0439 | 745.6886 | 3 |
|  | 99 | WLSSEIEDVQPAKTIPTSGP |  | missed K-T@13 | 0.0039 | 2154.0881 | 719.0367 | 3 |
|  | 99 | WLSSEIEDVQPAKTIPTSGP | Formyl(K)@13 | missed K-T@13 | 0.0025 | 2182.082 | 728.3679 | 3 |
|  | 99 | WLSSEIEDVQPAKTIPTSGP | FormaldehydeAdduct(W)@1;Formyl(K)@13 | missed K-T@13 | 0.006 | 2194.0854 | 732.3691 | 3 |
|  | 99 | WLSSEIEDVQPAKTIPTSGP | Carbamidomethyl(K)@13 | missed K-T@13 | -0.0039 | 2211.1021 | 738.0413 | 3 |
|  | 99 | YVVNHLGR |  |  | 0.0024 | 956.5217 | 479.2681 | 2 |
|  | 99 | YVVNHLGR | Carbamidomethyl(H)@5 |  | 0.0015 | 1013.5423 | 507.7784 | 2 |
|  | 99 | YVVNHLGR |  |  | 0.0024 | 956.5217 | 479.2681 | 2 |
| 3^D^ | 99 | AVEHSSVVINLVGR |  |  | -0.0031 | 1478.8174 | 493.9464 | 3 |
|  | 99 | AVGEKEVR | Formyl(K)@5 | missed K-E@5 | -0.0035 | 914.4787 | 458.2466 | 2 |
|  | 99 | ETFPEATIIKPAEIFGREDR |  | missed R-E@17 | 0.0048 | 2318.1958 | 580.5562 | 4 |
|  | 99 | FLNYFANIR |  |  | -0.0004 | 1156.6024 | 579.3085 | 2 |
|  | 99 | GIINAIKDPDAR |  | missed K-D@7 | 0.0018 | 1281.7059 | 641.8602 | 2 |
|  | 99 | GIINAIKDPDAR | Formyl(K)@7 | missed K-D@7 | 0.0029 | 1309.7019 | 655.8582 | 2 |
|  | 99 | GSQVIVPHR |  | cleaved M-G@N-term | -0.0019 | 991.5544 | 496.7845 | 2 |
|  | 99 | IPQAIAQVSK |  |  | 0.0001 | 1053.6185 | 527.8165 | 2 |
|  | 99 | MGSQVIVPH |  | cleaved H-R@C-term | -0.0043 | 966.4915 | 484.253 | 2 |
|  | 99 | MGSQVIVPH | Oxidation(M)@1 | cleaved H-R@C-term | 0.0029 | 982.4935 | 492.254 | 2 |
|  | 99 | MGSQVIVPHR | Oxidation(M)@1; Carbamidomethyl(H)@9 |  | -0.001 | 1195.6123 | 598.8134 | 2 |
|  | 99 | MGSQVIVPHR | Phospho(S)@3 |  | -0.007 | 1202.556 | 602.2853 | 2 |
|  | 99 | MGSQVIVPHR | Oxidation(M)@1 |  | -0.0019 | 1138.5898 | 1138.5898 | 2 |
|  | 99 | MGSQVIVPHR |  |  | 0.0003 | 1122.597 | 562.3058 | 2 |
|  | 99 | QPVYIVDVTK |  |  | 0.001 | 1160.645 | 581.3298 | 2 |
|  | 99 | QPVYIVDVTK | Gln->[pro-Glu@N-term](mailto:pro-Glu@N-term); Cation:Na(D)@7 |  | -0.0017 | 1165.5979 | 583.8062 | 2 |
|  | 99 | QPVYIVDVTK | Cation:Na(D)@7 |  | -0.0006 | 1182.6255 | 592.32 | 2 |
|  | 99 | QPVYIVDVTK | Gln->pyro-Glu@N-term |  | -0.0005 | 1143.6171 | 572.8158 | 2 |
|  | 99 | SKAVGEKEVR | Formyl(K)@2;  Formyl(K)@7 | missed K-A@2; missed K-E@7 | -0.002 | 1157.6021 | 579.8083 | 2 |
|  | 99 | SSSKYLR | Formyl(K)@4 | missed K-Y@4 | -0.0031 | 867.4419 | 434.7282 | 2 |
|  | 99 | SSVSGIVATVFGATGFLGR |  |  | -0.0005 | 1824.973 | 609.3316 | 3 |
|  | 99 | TFAFVGPSR |  |  | -0.0013 | 980.5067 | 491.2606 | 2 |
|  | 99 | TVKQPVYIVDVTK | Phospho(T)@1 | missed K-Q@3 | -0.0085 | 1568.8131 | 785.4138 | 2 |
|  | 99 | TVKQPVYIVDVTK |  | missed K-Q@3 | 0.0004 | 1488.8553 | 497.2925 | 3 |
|  | 99 | TVKQPVYIVDVTK | Formyl(K)@3 | missed K-Q@3 | 0.0035 | 1516.8536 | 759.4341 | 2 |
|  | 99 | WLSSEIEDVQPAK | Dioxidation(W)@1 |  | 0.0032 | 1532.739 | 767.3768 | 2 |
|  | 99 | WLSSEIEDVQPAK | Phospho(S)@4 |  | -0.0091 | 1580.7032 | 791.3589 | 2 |
|  | 99 | WLSSEIEDVQPAK |  |  | -0.0004 | 1500.7457 | 751.3801 | 2 |
|  | 99 | WLSSEIEDVQPAK | FormaldehydeAdduct(W)@1 |  | 0.0033 | 1512.7495 | 757.3819 | 2 |
|  | 99 | YVVNHLGR |  |  | -0.0013 | 956.5179 | 479.2662 | 2 |
|  | 99 | YVVNHLGR | Carbamidomethyl(H)@5 |  | 0.0001 | 1013.5406 | 507.7776 | 2 |

^a^ Refers to the spots excised for protein identification; superscripts following the numbers stand for A-form (A) and D-form (D).
